# Downregulated NPAS4 in multiple brain regions is associated with major depressive disorder

**DOI:** 10.1038/s41598-023-48646-9

**Published:** 2023-12-07

**Authors:** Berkay Selçuk, Tuana Aksu, Onur Dereli, Ogün Adebali

**Affiliations:** 1 Molecular Biology, Genetics and Bioengineering Program, Faculty of Engineering and Natural Sciences, Sabanci University, 34956 Istanbul, Turkey; 2 TÜBİTAK Research Institute for Fundamental Sciences, 41470 Gebze, Turkey

**Keywords:** Computational biology and bioinformatics, Genetics, Neuroscience, Psychology

## Abstract

Major Depressive Disorder (MDD) is a commonly observed psychiatric disorder that affects more than 2% of the world population with a rising trend. However, disease-associated pathways and biomarkers are yet to be fully comprehended. In this study, we analyzed previously generated RNA-seq data across seven different brain regions from three distinct studies to identify differentially and co-expressed genes for patients with MDD. Differential gene expression (DGE) analysis revealed that NPAS4 is the only gene downregulated in three different brain regions. Furthermore, co-expressing gene modules responsible for glutamatergic signaling are negatively enriched in these regions. We used the results of both DGE and co-expression analyses to construct a novel MDD-associated pathway. In our model, we propose that disruption in glutamatergic signaling-related pathways might be associated with the downregulation of NPAS4 and many other immediate-early genes (IEGs) that control synaptic plasticity. In addition to DGE analysis, we identified the relative importance of KEGG pathways in discriminating MDD phenotype using a machine learning-based approach. We anticipate that our study will open doors to developing better therapeutic approaches targeting glutamatergic receptors in the treatment of MDD.

## Introduction

Major Depressive Disorder (MDD), also known as depression, is a common psychiatric disorder that affected more than 2% of the world population (163 million people) in 2017^1^. It is characterized by low mood sustained for at least 2 weeks, often with low self-esteem, loss of interest in normally enjoyable activities, low energy, and pain without a clear cause. Among more severe symptoms, suicidal behaviors are observed in patients with major depression, making it one of the most common fatal disorders in the world^2^. Recently, the severe depression rate among youth escalated from 9.4 to 21.1% between 2013 and 2018^3^. This suggests a rising trend in the number of depressive patients and emphasizes the importance and urgency of the problem. Therefore, immediate research is needed to define fine-established markers of major depression to address this ongoing global well-being problem.

Several attempts have been made to identify the transcriptional profiles of patients with major depression by using next-generation sequencing (NGS) data obtained from postmortem patients. Pantazatos et al.^4^ have discovered thirty-five differentially expressed genes in the dorsolateral prefrontal cortex of depression sudden deaths (MDD) and depression suicidals (MDD-S) compared to the control group (padj < 0.1). The DLPFC is implicated in regulating impulsivity, decision-making, cognitive control of mood and other tasks related to suicidal tendencies^5,6^. However, only a single brain region, with a limited sample size of 59, was investigated in that study.

Labonté et al.^7^ examined six brain regions including DLPFC, nucleus accumbens (nACC), ventral subiculum (vSUB), anterior insula (aINS), cingulate gyrus 25 (Cg25), and orbitofrontal cortex (OFC). The nACC is integral to the reward system and has been a target for investigation of chronic stress and depression^8^. The vSUB acts as a modulator of the hippocampus that has been associated with depression^9^. The aINS is key for perceiving internal states and subjective emotions^10,11^, but there's no consensus on its association with MDD. The Cg25 plays a pivotal role in regulating emotions and guiding behavior^12^. Imaging studies in patients with depression have shown increased subcallosal cingulate gyrus activity, which may be reversed via antidepressant treatment^13^. The OFC is pivotal for emotion and evaluating reward value across various stimuli, distinguishing between expected rewards and non-rewards. An imbalance in reward and non-reward processing at the OFC, marked by altered activity and connectivity, plays a crucial role in the manifestation and treatment of depression^14^. Labonte et al. showed differences in transcriptional patterns of men and women in these regions, proposing sexual dimorphism for depression. Although researchers have discovered a 5–10% overlap for the differentially expressed genes for the females and males, the data did not yield any outstanding common genetic marker associated with MDD.

Similarly, in 2017, Ramaker et al.^15^ investigated transcriptional profiles of patients with schizophrenia, bipolar disorder, and major depression by using brain regions DLPFC, nACC and anterior cingulate (AnCg). While fundamental cognitive processes such as motivation, decision-making, learning, cost–benefit calculation, as well as conflict and error monitoring are associated with the AnCg^12^, there's no clear linkage to MDD yet. Although they have identified differentially expressed genes (padj < 0.05) for schizophrenia and bipolar disorder, they have not identified any for major depression. Sequencing data from these three valuable studies can be analyzed together to increase the sample size and improve the resolution of the results.

In this study, we combined and analyzed previously used RNA-seq data from multiple studies^4,7,15^ to identify genes that are differentially expressed for MDD by considering the factors of gender, age, postmortem interval, brain region, and the study they belonged to. We conducted differential gene expression analysis across seven distinct brain regions DLPFC, nACC, vSUB, aINS, AnCg, Cg25 and OFC. Out of these, only regions DLPFC, nACC and vSUB exhibited differential gene expression in patients with MDD. These three regions were further studied for significant gene expression changes and co-expressing gene modules. Lastly, used a non-linear, machine learning based approach to determine biological pathways that can be used for diagnostic purposes by using samples from all brain regions. We present significant genetic biomarkers and pathways associated with the major depression phenotype.

## Results

We combined RNA-seq datasets from three different sources^4,7,15^ containing sequenced brain tissue samples from postmortem control and major depression patients to identify statistically significant transcriptional changes. We analyzed the raw RNA sequencing reads and measured the expression levels of genes for each sample. The quality of each sample was assessed, and a few samples were discarded from the analysis due to having low quality (see "Methods"). Then, we followed the general pipeline of RNA-seq data analysis (see "Methods") by performing alignment to the human genome and counting the reads aligned with each gene. We grouped the counts according to the brain region they belonged to and identified genes that are differentially expressed relative to the control group (padj < 0.05) for each region by using the DESeq2 R package^16^. We did not apply any log-fold change cut-off to our analysis.

**Figure 1:**
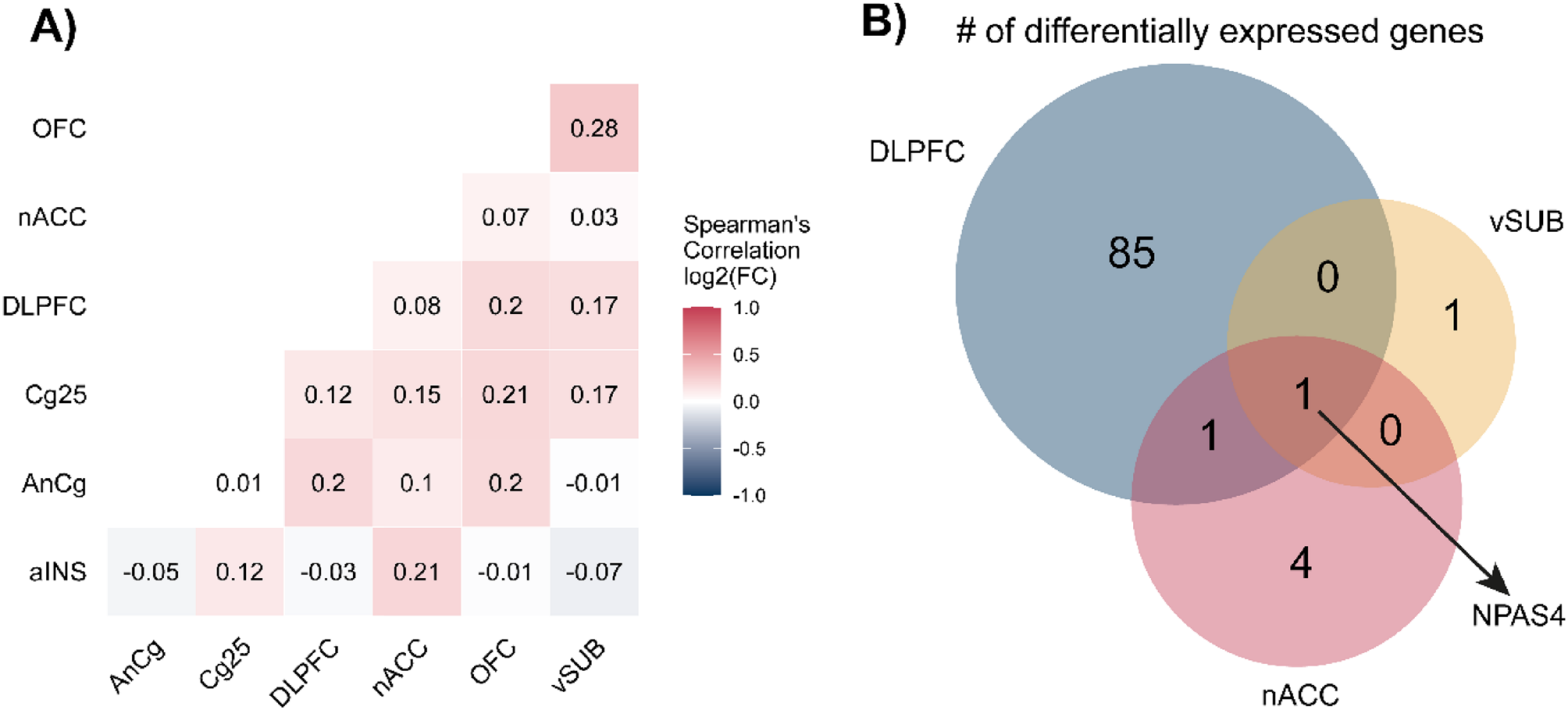
Differential gene expression analysis for different brain regions (**A**) Spearman’s correlation of log2FC values between investigated brain regions. (**B**) Venn diagram showing the number of differentially expressed genes for DLPC, vSUB, and nACC.

To investigate the potential transcriptional similarities between different brain regions, we first calculated pairwise Spearman’s correlations using log2FC values of commonly expressed genes (Fig. 1A). No strong correlation was observed between the two regions. The strongest correlation was observed between the orbitofrontal cortex and ventral subiculum with pairwise Spearman’s correlation score of 0.28. Therefore, we can conclude that different disease-related signatures were observed in different brain regions.

Then, we focused on genes that are differentially expressed for each region independently. Out of the seven regions, we identified at least one differentially expressed gene in DLPFC, nACC, and vSUB but not in other brain regions. The highest number of differentially expressed genes was observed in DLPFC (sample size of n = 150) with 87 differentially expressed genes, and this was followed by nACC (n = 94) with six genes and vSUB (n = 43) with two genes (Fig. 1B). When we intersected lists of differentially expressed genes for these three regions, we discovered that a brain-specific transcription factor NPAS4^17^ was the only common gene (Fig. 1b) that was downregulated in all three regions. It was previously shown in mice^18–21^ and in a study monitoring 152 ischemic stroke patients^22^ that decrease in NPAS4 expression is correlated with the MDD phenotype.

**Figure 2:**
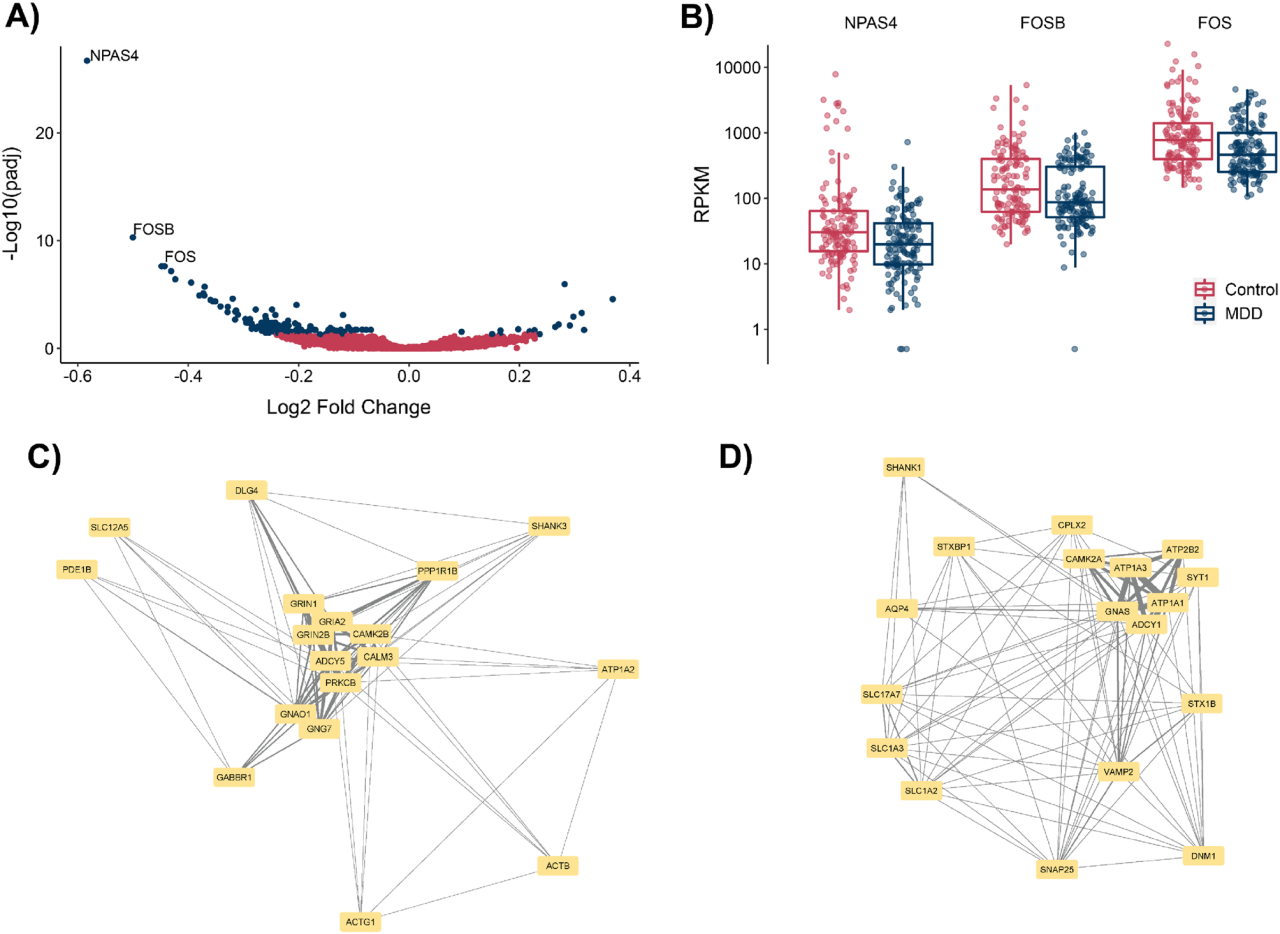
DGE and co-expression analysis of DLPFC, vSUB and nACC (**A**) Volcano plot for the DGE analysis of three regions. (**B**) Box plots for the top three differentially expressed genes NPAS4, FOSB, and FOS. (**C**, **D**) Top genes in glutamatergic signaling and synaptic vesicle cycle, and secretion co-expression modules enriched in KEGG pathways. Edges indicate co-occurrence in the same pathway. Higher edge width indicates higher co-occurrence. Force directed layout is used for visualization.

Because NPAS4 was identified as the single common downregulated gene, we aimed to further investigate the shared transcriptional profile between different regions. Therefore, we combined samples from three regions (DLPFC, nACC, and vSUB) which we observed differential gene expression and reached a sample size of 287 (143 CTRL, 144 MDD) to perform a DGE analysis by adding a covariate of “brain region” to eliminate region-specific variations in gene expression. As presented in the volcano plot (Fig. 2A), 149 genes were found to be differentially expressed (padj < 0.05) with a general trend of downregulation. We suggest that this was mainly due to the top three (padj:2 × 10^–27^, 4.9 × 10^–11^, 2.3 × 10^–8^) downregulated transcription factors (NPAS4, FOS, and FOSB) (Fig. 2B).

To gain more insight into the pathways involved in MDD phenotype, we performed a co-expression analysis using CEMiTool^23^ for the brain regions we observed NPAS4 downregulation to reveal correlating gene modules. As an input, we used the same normalized count matrix for DGE analysis. The co-expression analysis yielded two co-expressed gene modules (padj < 0.1) as modules 1 and 2. After introducing sample annotations as MDD and control, we identified that both of the modules show positive enrichment in control patients and negative enrichment in MDD patients (Supplementary Fig. 1). For the first module (128 genes) control group had normalized enrichment score (NES) of 1.49 (padj = 0.048) and MDD group had -1.48 (padj = 0.036). Furthermore, for the second gene (60 genes) module control group had NES of 1.42 and MDD group had −1.44 (padj = 0.065). Overall, higher enrichment means a higher activity of the module for a given group and the opposite is true for the negatively enriched group. Because the activity of each module is correlated with the expression levels of the samples, we can conclude that co-expressed genes are mainly downregulated for patients with depression. Although we repeated the same analysis by including all samples, we couldn’t identify functionally relevant co-expressing modules. Therefore, we can conclude that NPAS4 downregulation is likely to be the driving force behind the changes observed in co-expression modules. We further explored the functional implications of these modules to understand their possible disease relevance.

We performed gene set enrichment analysis through a web-based tool Enrichr^24–26^, and presented the top 10 KEGG (Kyoto Encyclopedia of Genes and Genomes)^27–29^ pathways based on their combined score (Tables 1, 2 and 3) for differentially genes and co-expressed gene modules. Enrichment of differentially expressed genes yielded 18 pathways (padj < 0.05) related to inflammation, such as the IL17 signaling pathway, Rheumatoid arthritis, NF-kappa B signaling pathway. It has been previously suggested that IL-17A induces depressive behavior in mice^30,31^, but studies including human subjects^32–34^ have contradicting conclusions. Lui et al.^35^ showed that higher serum levels of IL-17 are positively correlated with the severity of anxiety in patients with rheumatoid arthritis. The involvement of interleukins and cytokines was previously discussed numerous times^36–38^. However, the genes highlighted in both these studies and our own analysis lack a strong association with the disorder. Therefore, they should be mainly considered as biomarkers. We suggest that a single pathway might be insufficient to capture the full breadth of disease biology; instead, contrasting multiple pathways can provide a more holistic perspective on observed patterns, in our case inflammation related genes. It should be noted that 91 out of 147 differentially expressed genes (including NPAS4) are not present in any KEGG pathway. This suggests that these pathways should not be considered representatives of all differentially expressed genes.

**Table 1:** KEGG 2021 pathway enrichment for the differentially expressed genes. Overlap: The overlap between the gene set and the pathway.

KEGG pathway	Overlap	Padj	Odds ratio	Combined score
IL-17 signaling pathway	9/94	5.20E-06	15.17	262.43
TNF signaling pathway	8/112	1.49E-04	10.93	144.84
Rheumatoid arthritis	7/93	3.18E-04	11.49	138.95
Legionellosis	5/57	0.001585773	13.41	129.97
Bladder cancer	4/41	0.0048795	14.98	125.53
AGE-RAGE signaling pathway in diabetic complications	7/100	3.86E-04	10.62	123.33
NF-kappa B signaling pathway	7/104	4.00E-04	10.18	115.59
Malaria	4/50	0.008858662	12.04	91.64
MAPK signaling pathway	10/294	0.001585773	5.03	48.47
Kaposi sarcoma-associated herpesvirus infection	7/193	0.008858662	5.29	39.48

**Table 2:** KEGG 2021 pathway enrichment for the glutamatergic signaling co-expression module (Module 1).

KEGG pathway	Overlap	Padj	Odds ratio	Combined score
Amphetamine addiction	8/69	1.04E-06	22.02	401.59
Cocaine addiction	6/49	1.79E-05	23.06	329.55
Circadian entrainment	9/97	1.04E-06	17.30	317.75
Glutamatergic synapse	9/114	2.51E-06	14.48	245.48
Gastric acid secretion	7/76	1.76E-05	16.88	244.71
Long-term potentiation	6/67	9.99E-05	16.24	201.68
Dopaminergic synapse	9/132	6.70E-06	12.35	193.68
GABAergic synapse	6/89	3.31E-04	11.92	128.40
Morphine addiction	6/91	3.44E-04	11.64	123.89
Oxytocin signaling pathway	8/154	1.30E-04	9.16	110.15

**Table 3:** KEGG 2021 pathway enrichment for the synaptic vesicle and secretion co-expression module. (Module 2).

KEGG pathway	Overlap	Padj	Odds ratio	Combined score
Synaptic vesicle cycle	10/78	2.72E-12	59.64	1870.16
Insulin secretion	7/86	3.41E-07	33.84	640.54
Endocrine and other factor-regulated calcium reabsorption	5/53	1.09E-05	38.37	558.02
Salivary secretion	6/93	1.09E-05	25.83	386.58
Aldosterone synthesis and secretion	6/98	1.09E-05	24.42	357.87
Gastric acid secretion	5/76	4.77E-05	25.91	329.90
Glutamatergic synapse	6/114	1.99E-05	20.79	286.04
Bile secretion	5/90	8.56E-05	21.63	257.31
Pancreatic secretion	5/102	1.42E-04	18.94	213.74
Vasopressin-regulated water reabsorption	3/44	0.003	26.00	211.24

We further investigated pathways enriched for individual co-expressed gene modules comparatively to reveal the significant patterns observed within modules. We called the first module as “glutamatergic signaling module” (Table 2) because we observed a strong enrichment for the addiction^39,40^ glutamatergic synapse, and circadian entrainment^41,42^ pathways that were mainly controlled by AMPA (α-amino-3-hydroxy-5-methyl-4-isoxazolepropionic acid) and NMDA (N-methyl-D-aspartate) glutamate receptor activity. To visualize the important co-expressed genes in pathways, we constructed a network visualized in Fig. 2C. In this visual presentation, we added edges between genes based on their co-occurrence in top 9 KEGG. Here, genes with high co-occurrence are clustered closely. Notably, glutamate receptors GRIN1, GRIN2B, GRIA2, Ca^2+^ dependent CALM3, CAMK3B, PRKCB, ADCY5, and G proteins GNAO1, GNG7 are form the core of this network. Lastly, we called the second module “synaptic vesicle and secretion module” (Fig. 2D and Table 3) because it contained genes ATP2B2 responsible for Ca^+2^ secretion, ATP1A3, ATP1A1 responsible for Na^+^/K^+^ transport, CAMK2A associated with Ca^+2^, GNAS and ADCY1 associated with G proteins. Therefore, the synaptic vesicle cycle, different secretion-related pathways, and pathways related to calcium transport are highly enriched. Negative enrichment scores of these two modules for the MDD suggest that glutamatergic signaling activity is downregulated for the brain regions where we observed NPAS4 as a common downregulated gene. This suggests a sequence of events leading NPAS4 downregulation. Previous research has highlighted the significance of both glutamatergic signaling and Ca^2+^ release in regulating NPAS4 induction^43^. Our findings postulate that the downregulation of glutamatergic pathways may trigger a dysregulation in Ca^2+^ transport resulting in to decreased NPAS4 expression and activity. NPAS4 has been involve in controlling the expression of genes linked to glutamatergic synapses, suggesting a reciprocal relationship between NPAS4 and these synapses^44^. The downregulation of these genes and pathways can result in reduced synaptic plasticity leading MDD^45^. Interestingly, when we extended the co-expression analysis across all brain regions in our study, we failed to reveal a significant pathway enrichment, reinforcing the unique association between NPAS4 and the identified pathways.

While DGE and co-expression analyses provided important insights about the changes in observed MDD patients, they are specifically designed to identify linear associations observed in expression data for pre-determined conditions (e.g., disease and control). However, in reality, disease biology can be much more complex that these analyses may not capture the true essence of the changes especially for the diseases such as MDD. Thus, in addition to DGE and co-expression analyses, we adopted a machine learning-based approach called multiple kernel learning (MKL). This method is specifically designed to capture non-linear relationships between genes and gene groups, helping identify disease-associated biological mechanisms. Notably, the same computational framework was shown to be successful in identifying features that predict cancer stages^46^ and the survival of individuals^47^. In our analysis, KEGG pathways were used to identify the informative gene groups to discriminate MDD patients from the control group. In this method, each pathway was mapped to a gene expression matrix, and distinct kernel matrices were calculated for each pathway. Using the optimized weighted combination of these kernel matrices, the algorithm finds a sparse set of pathways by discarding uninformative ones from the collection. We can infer the relative importance of the pathways by considering their resulting kernel weights. We used the normalized gene expression values from all brain regions and samples (n = 457) to identify the common underlying biological mechanisms associated with MDD.

**Figure 3:**
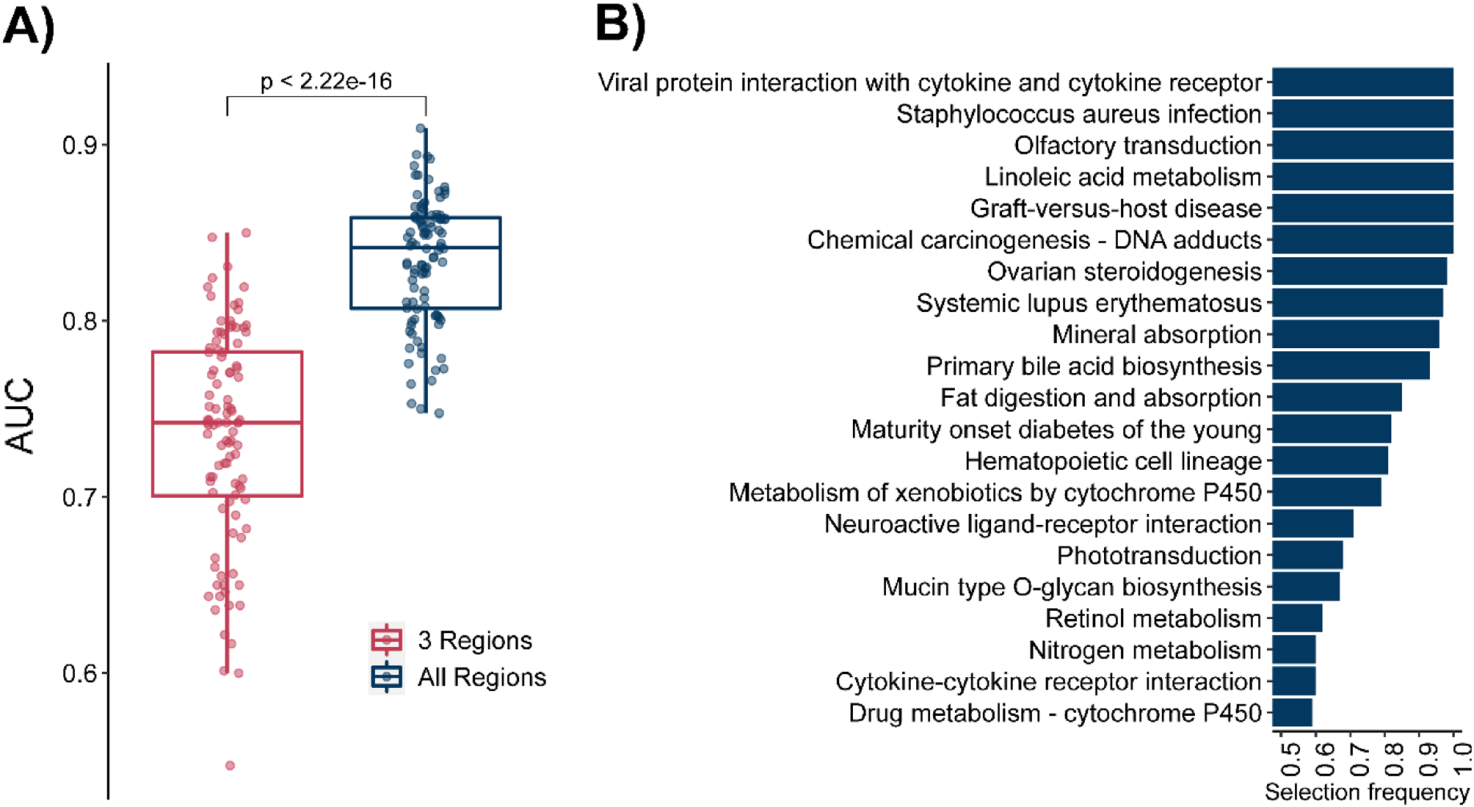
Multiple kernel learning results. (**A**) Area under curve comparison of multiple kernel learning for 100 replications. (**B**) Pathways selected as discriminative in 100 replication more than 50 times.

We reported the area under the receiver operating characteristic curve (AUC) values over 100 replications to evaluate the algorithm’s performance. The predictive performance of the MKL algorithm is increased when we included samples from all regions compared to three regions containing differentially expressed genes (Fig. 3A) indicating that including more brain regions and samples in the analysis increases the reliability of the prediction model. We achieved an average AUC score of 0.84 with a standard deviation of 0.04 for the model including all brain regions (Fig. 3A). 21 pathways were selected as informative, at least in 50 replications (Fig. 3B). Pathways “Linoleic acid metabolism,” “Viral protein interaction with cytokine and cytokine receptor,” “Olfactory transduction,” “Staphylococcus aureus infection,” “Chemical carcinogenesis—DNA adducts,” and “Graft-versus-host disease” were selected as informative in all replicates. Because some of the chosen pathways do not directly relate to brain tissue, we would like to elaborate on the results by categorizing them based on the gene groups they share. Thus, we divided pathways into two main categories based on their functional relevance and gene composition. The first cluster contained eight pathways (Linoleic acid metabolism, Chemical carcinogenesis—DNA adducts, Ovarian steroidogenesis, Primary bile acid biosynthesis, Fat digestion and absorption, Maturity onset diabetes of the young, Metabolism of xenobiotics by cytochrome P450, Retinol Metabolism, and Drug metabolism—cytochrome P450) containing genes related to synthesis, absorption, and metabolism of lipids. In this group, genes related to the cytochrome p450 (CYP) family are abundant and shared between different pathways. Previous studies have focused on variants in CYP genes and their association with SSRI metabolism and the effectiveness of the treatment^48–51^ On the other hand, our approach puts forward the idea that they can be used for diagnosis. “Nitrogen metabolism” and “Maturity onset diabetes of the young” can also fit in this category because they are related to metabolism. Several studies^52,53^ demonstrate the role of metabolism in patients with MDD. The second major group contained five pathways (Viral protein interaction with cytokine and cytokine receptor, Staphylococcus aureus infection, Graft-versus-host disease, Hematopoietic cell lineage, and Cytokine-cytokine receptor interaction) related to inflammation and immune system which is parallel to the enrichment of differentially expressed genes that we identified. The remaining four pathways were related to perceiving external stimuli through receptors (Olfactory transduction, Neuroactive ligand-receptor interaction, and Phototransduction) and glycosylation (Mucin type O-glycan biosynthesis). Overall, using KEGG pathways as features, we discriminated against MDD patients with high accuracy. The pathways we identified as discriminative can serve as a starting point for the research on MDD diagnosis.

## Discussion

Our study combined multiple publicly available RNA-Seq datasets to identify novel pathways and genetic markers associated with MDD. A large sample size increased the sensitivity of the analysis, which led to the discovery of novel gene-disease associations. On the other hand, combining datasets from different sources introduces a certain amount of noise to the analysis. Moreover, Brodmann areas, individual segments of the cerebral cortex defining boundaries of each brain region, for a given region might slightly differ between studies. Therefore, we performed a preliminary quality filtration step and used the “study” covariate in our DGE analysis to eliminate some of that noise.

Our results show that the dorsolateral prefrontal cortex is the most affected region based on the number of differentially expressed genes, and downregulation of NPAS4 is observed for multiple brain regions. It should be highlighted that the larger change observed in DLPFC can also be attributed to its larger sample size. It has been previously demonstrated that NPAS4 plays a role in memory^54^, modulating inhibitory-excitatory balance^55–57^, epileptogenesis in mice, cocaine-induced hyperlocomotion^43^, cognitive well-being and many other diseases^19,58,59^. While the association between NPAS4 and MDD has been shown in mice previously^20^, we validated the same relationship for humans and multiple brain regions. Supporting our findings, Gu et al. showed that patients with post-stroke depression had lower expression levels of NPAS4 in their peripheral blood mononuclear cells^22^, which makes NPAS4 a potential diagnostic biomarker in the future. Our study suggests the central role of NPAS4 in major depression as an association factor. Although this study suggests a potential causation role of NPAS4 in the downregulation of synaptic plasticity in MDD, this hypothesis needs to be tested experimentally in model species.

**Figure 4:**
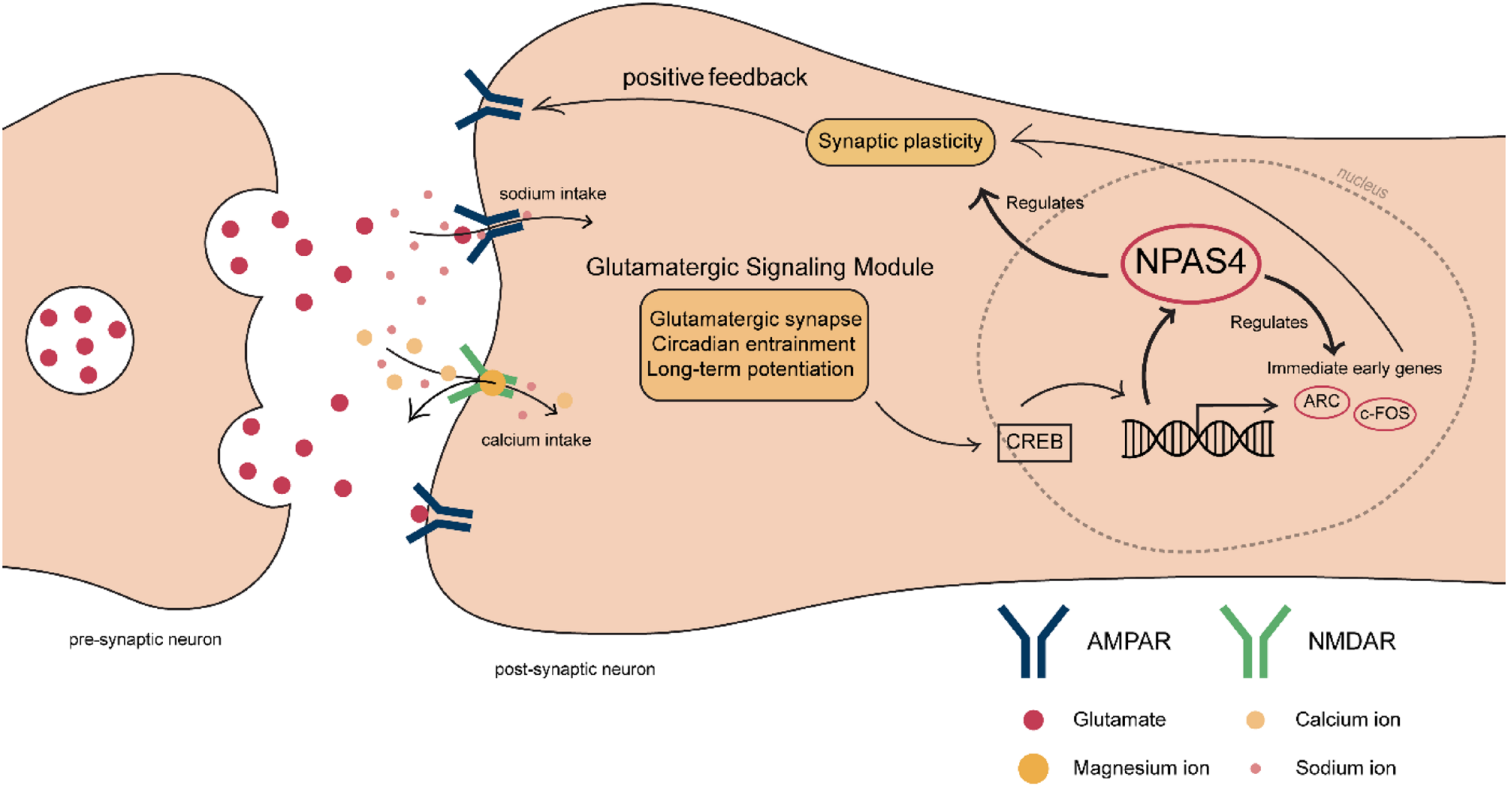
Summary of differentially and co-expressed genes and the enriched pathways.

To highlight the role of NPAS4 and understand that the relationship between differentially genes and co-expressed gene modules, we gathered our findings into an MDD model (Fig. 4). In this model, we combined our findings with the existing literature on connections between genes and pathways. In our model, we show that NMDA and AMPA glutamate receptors can induce the expression of NPAS4 and other IEGs through controlling Ca^2+^ influx^60,61^. Previous research points to a synergistic interaction between glutamatergic synapses and NPAS4, where both amplify each other's activity to increase synaptic plasticity^43,44^. ChIP-seq enhancer data of NPAS4 within mouse cortical neurons show that NPAS4 regulates immediate early genes^62^. Differentially expressed genes and this experiment include IEGs in common; FOS, FOSB, NR4A1, NR4A3, JUNB, and NPAS4 itself. ChIP-seq data of NPAS4 embryonic mouse 14 days medial ganglionic eminence (mostly containing excitatory neurons) and cortex (mostly inhibitory neurons) shows that NPAS4 regulates distinct sets of late-response genes in inhibitory and excitatory neurons^57^. Genes identified in our DGE analysis PTGS2, ATF3, ETV3, and CSRPN1 which were also regulated by NPAS4 in inhibitory neurons. By controlling the expression of other IEGs, which are also transcription factors, NPAS4 indirectly regulates the expression of many different genes as a master transcription factor controlling synaptic plasticity. Supporting our model, existing studies have shown that FOS, FOSB, and their splice variants^63–65^ are associated with motivation and depressive behavior^66^. Also, some antidepressants have been shown to increase the expression of FOS^67^ and NPAS4^68^. These immediate early genes play important roles in maintaining essential synaptic functions^69–71^. In our analysis, we observed a significant downregulation trend in pathways regulated by glutamatergic receptors. These pathways influence IEGs that regulate synaptic plasticity, circadian entrainment^72–74^, and learning abilities (long-term potentiation) in patients with MDD.

Building on the insights from our model which highlights the significance of glutamatergic receptors in the context of MDD, it's worth revisiting existing therapeutic strategies. Traditionally, the focus has been on aminergic receptors, especially serotonin and dopamine^75^. However, these monoamine-oriented treatments have been ineffective, especially for patients with treatment-resistant depressions^76,77^. Our study suggests that glutamatergic receptors can be used as drug targets in the treatment of MDD, aiming to restore the lost synaptic plasticity. In support of our hypothesis, NMDA antagonist ketamine and its enantiomer esketamine have been shown to be effective for patients with treatment resistant MDD^76,78–80^. Although esketamine is an antagonist of the NMDA receptor, it leads to the activation of AMPA receptors^81^ that increase synaptic plasticity. Furthermore, the trial of NMDA co-agonist glycine triggered depressive symptoms in mice^82^. Thus, we conclude that the results of these drug trials align with the model we proposed in this study. We anticipate that antidepressants targeting glutamatergic signaling pathways will gain more popularity.

## Materials and methods

### Datasets

In this study, three postmortem RNA-seq datasets from Gene Expression Omnibus (GSE101521, GSE80655, and GSE102556)^4,7,15^ were combined to increase the sample size and perform a statistically significant analysis of the MDD profile. A total of 216 control (28.70% female) and 241 major depressive disorder samples (42.74% female) were investigated based on their gene expression profiles (Table 4). The average age of death of CTRL and MDD samples are 47.66 and 46.78, respectively. Samples from 7 brain regions, including the dorsolateral prefrontal cortex (DLPFC), nucleus accumbens (nACC), ventral subiculum (vSUB), anterior insula (aINS), anterior cingulate cortex (AnCg), cingulate gyrus 25 (Cg25), and orbitofrontal cortex (OFC) were analyzed (Table 5).

**Table 4:** Demographics of study groups.

	Control	MDD
Sample size	N = 216	N = 241
Age (years, avg)	47.66 (sd = 15.18)	46.78 (sd) = 15.21)
Gender (male–female)	154–62	138–103
PMI (hours, avg)	24.08 (sd = 16.22)	26.32 (sd) = 16.24)

**Table 5:** Distribution of samples by brain regions and the study groups that they belong to.

Brain region	Control (N)	MDD (N)	Total
Anterior cingulate cortex (AnCg)	24	23	47
Anterior insula (aINS)	22	26	48
Cingulate gyrus 25 (Cg25)	15	13	28
Dorsolateral prefrontal cortex (DLPFC)	71	79	150
Nucleus accumbens (nACC)	43	51	94
Orbitofrontal cortex (OFC)	22	25	47
Ventral subiculum (vSUB)	29	24	43

## Data analysis

### Quality trimming

FASTQC 0.11.7 was used to check the quality of each sample. We eliminated some of the samples directly from the analysis due to having very low quality in general. For the samples having low quality towards the 3’ end, we used Cutadapt^83^ with the “–quality-cutoff 10” option. After performing 3’ trimming we concatenated fasta files for each patient when there are multiple fasta files for a single patient.

### Alignment to the human genome

TopHat 2.1.1^84^ was used for aligning reads to the human genome (GRCh37)^85^. At this step, we converted fasta files into bam files. Then by using the samtools^86^ sort option we converted bam files to sam.

### Read count

HTSeq^87^ was used to obtain read counts for each patient. The distribution of counts for each region is given in Fig. 1. Ensembl GRCh37 annotation list was used as a reference.

### Differential gene expression analysis

Differential gene expression analysis based on the negative binomial distribution was performed in R with DESeq2 package^16^. Genes that significantly differentially expressed (adjusted *p*-value < 0.05) between major depressive patients and the control group were identified regarding sex, age, study and brain region that the sample is obtained from, and postmortem interval covariates (full model, design ~ sampleDataset + sampleGender + sampleAge + PMI + brainRegion + condition; brain region-specific model, design ~ sampleDataset + sampleGender + sampleAge + PMI + condition).

### Co-expression analysis

R package CEMiTool^23^ was used to perform co-expression analysis. Normalized count data from DLPFC, vSUB and nACC were included in the analysis. Variance stabilizing transformation was not applied before filtering the genes and default filtering p-value was used (0.1). Label of each sample was provided to obtain normalized enrichment scores for each of the modules in control group and MDD patients.

### Identification of MDD-associated pathways using MKL algorithm

A multiple kernel learning (MKL)-based machine learning approach^46^ was used to identify informative pathways in discriminating MDD patients. Instead of first identifying the expressed genes and then performing a gene set enrichment analysis using these selected genes, the proposed MKL-based algorithm considers whole expression matrix and each pathway from the given collection at the same time. In this method, each pathway is mapped to a different kernel function using the expression profiles of the genes in the given pathway. Kernel functions are defined as the similarity measures between pairs of samples, and it is known that weighted combination of several kernel functions (i.e., MKL) increases the predictive ability of the kernel-based methods^88^. At the end, the proposed method converges to a solution where kernels with non-zero weights are included in the final model for the classification. We considered that a pathway is selected to be used in the final model if the corresponding kernel weight was greater than 0.01.

The experimental setting that we used in machine learning model is as follows. We split our dataset by randomly picking 80% as training and 20% as test set. While splitting the data, we kept the ratio between the control group and MDD patients same in the training and test partitions. We repeated this procedure 100 times to obtain more robust performance measures and reported the experimental results over these 100 replications. We performed fourfold inner cross-validation for selecting the model parameters (i.e., regularization parameter C). Since the gene expression is a normalized count data, we first log2-transformed our dataset. Following that, we further normalized the training set to have zero mean and unit standard deviation, while we normalized the test set using the mean and the standard deviation of the original training set. We followed the same computational setting as proposed in^46^ to obtain the relative importance of pathways.

### Supplementary Information

Supplementary Information.

## Data Availability

The open-source code and supplementary data are available at our GitHub repository: https://github.com/CompGenomeLab/mdd-analysis.
