# Supplementary material for: Downregulated NPAS4 in multiple brain regions is associated with major depressive disorder
Source: Sci Rep. 2023 Dec 7;13:21596. doi: 10.1038/s41598-023-48646-9 (PMC10703936; doi:10.1038/s41598-023-48646-9)
Supplement: Supplementary file 1 — Supplementary Information. [file 41598_2023_48646_MOESM1_ESM.zip › mdd-analysis-github-contents/analysis/R-code.pdf]

Differential Gene Expression (DGE) Analysis

- Import sample table as data.frame

```
#This dataframe contains
sampleTable <- data.frame(sampleName = sampleName,fileName = sampleFiles,condition = sampleCondit
View(sampleTable)
```

sampleName	fileName	condition	sampleGender	sampleAge
SRR5831944	SRR5831944_count_control_1_m.txt	CTRL	male	17
SRR5831945	SRR5831945_count_control_1_f.txt	CTRL	female	30
SRR5831946	SRR5831946_count_control_1_m.txt	CTRL	male	66
SRR3438661	SRR3438661_count.txt	CTRL	male	32
SRR3438672	SRR3438672_count.txt	CTRL	male	66
SRR3438673	SRR3438673_count.txt	CTRL	male	49

```
# Formation of data frame and converting condition, dataset, gender, brain region informations in
sampleTable$condition <- factor(sampleTable$condition)
sampleTable$sampleDataset <- factor(sampleTable$sampleDataset)
sampleTable$sampleGender <- factor(sampleTable$sampleGender)
sampleTable$brainRegion <- factor(sampleTable$brainRegion)
```

```
# Importing DESeq2 library and forming dds object
library("DESeq2")

# We used gender, age, brain region, post mortem interval, and which datasets these samples belon
dds <- DESeqDataSetFromHTSeqCount(sampleTable = sampleTable,directory = directory,design= ~ sampl
dds <- DESeq(dds)
results <- results(dds)

# To find differentially expressed genes padj lower than 0.05 between MDD and control groups
contrast <- c("condition","MDD","CTRL")
res_tableOE_unshrunken <- results(dds, contrast=contrast, alpha = 0.05)
res_tableOE <- lfcShrink(dds, contrast=contrast, type = "normal",  res=res_tableOE_unshrunken)
res_tableOE_df <- data.frame(res_tableOE)
```

```
#adding a column for ensembl gene ids to the dataframe and removing rownames
res_tableOE_df$ensembl_gene_id <- rownames(res_tableOE_df)
rownames(res_tableOE_df) <- NULL

#Filtering of the results data frame based on our padj cutoff. After that we imported that data f
padj.cutoff <- 0.05
sig <- subset(res_tableOE_df, padj <= padj.cutoff)
write.csv(sig, "output directory here")
```

Co-expression Analysis

- Import sample annotation list

SampleName	Class
SRR5831944	CTRL
SRR5831945	CTRL
SRR5831958	MDD
SRR5831959	MDD

```
#Normalized counts are retrieved from dds. Counts are already normalized, thus "normalized=TRUE".
#We used same samples that we used for three region analysis.

normalized_counts <- counts(dds, normalized=TRUE)
normalized_counts <- as.data.frame(normalized_counts)

#Co-expression analysis
library("CEMiTool")
cem <- cemitool(normalized_counts, sample_annot)
generate_report(cem)
write_files(cem)
```
