# Supplementary material for: Downregulated NPAS4 in multiple brain regions is associated with major depressive disorder
Source: Sci Rep. 2023 Dec 7;13:21596. doi: 10.1038/s41598-023-48646-9 (PMC10703936; doi:10.1038/s41598-023-48646-9)
Supplement: Supplementary file 1 — Supplementary Information. [file 41598_2023_48646_MOESM1_ESM.zip › mdd-analysis-github-contents/cemitools_report.html]

CEMiTool


Code 

- Show All Code
- Hide All Code

# CEMiTool

# Report

## Modules

## Profile Plot

## Gene Set Enrichment Analysis

## Over Representation Analysis

### Please run over representation analysis!

## Interaction Network

### Please add interactions to the CEMiTool object

## Parameters
