# Supplementary material for: Downregulated NPAS4 in multiple brain regions is associated with major depressive disorder
Source: Sci Rep. 2023 Dec 7;13:21596. doi: 10.1038/s41598-023-48646-9 (PMC10703936; doi:10.1038/s41598-023-48646-9)
Supplement: Supplementary file 1 — Supplementary Information. [file 41598_2023_48646_MOESM1_ESM.zip › mdd-analysis-github-contents/Supplemetary Figures.pdf]

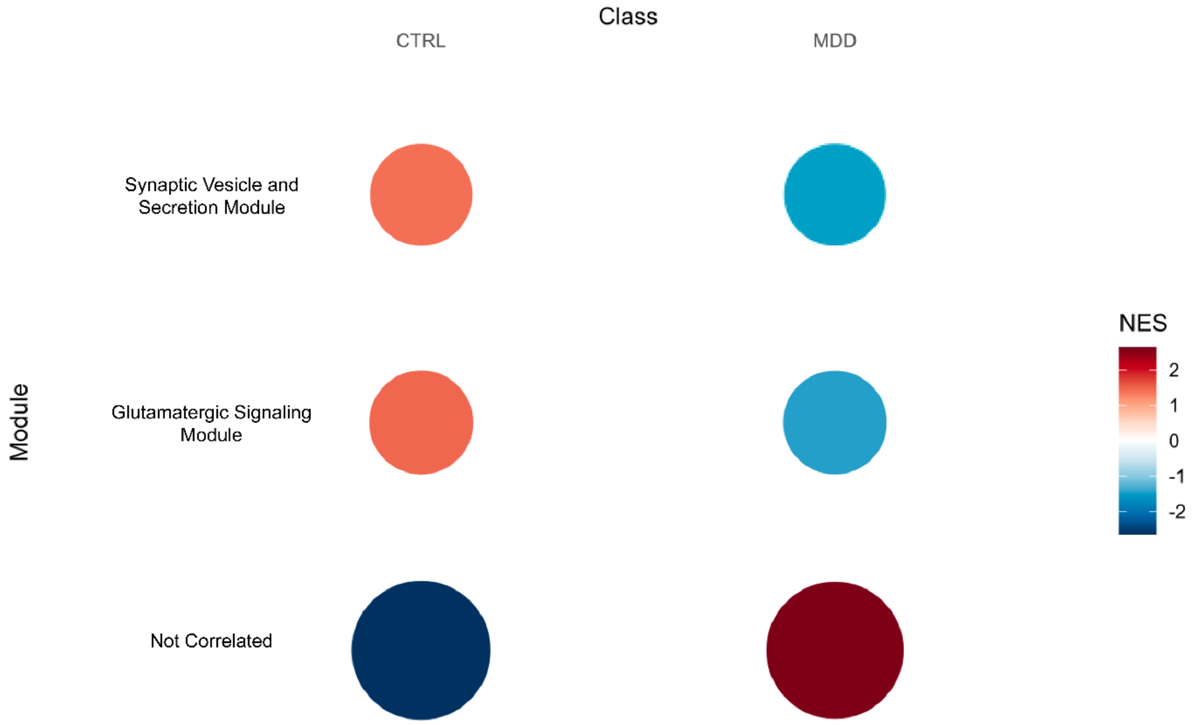

Supplementary Figure 1: Normalized enrichment scores (NES) for co-expressing gene modules in regions DLPFC, nACC, and vSUB.

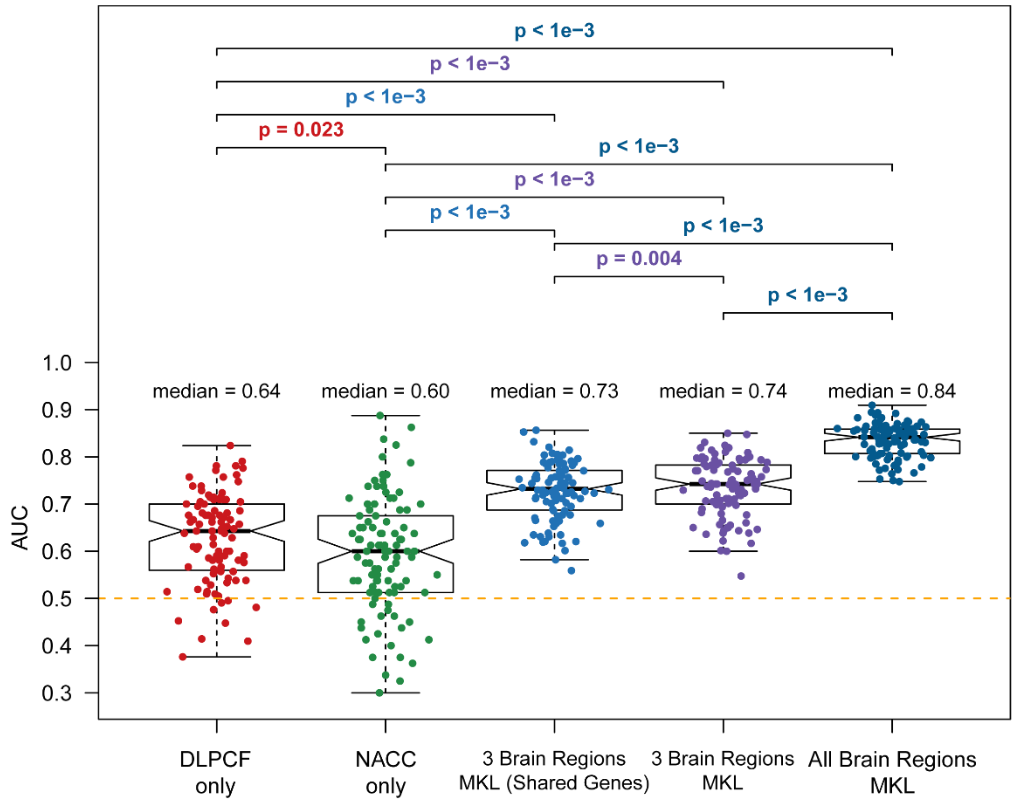

Supplementary Figure 2: Area under curve scores for the MKL algorithm for different sample combinations.
